# Paenidigyamycin A, Potent Antiparasitic Imidazole Alkaloid from the Ghanaian *Paenibacillus* sp. DE2SH

**DOI:** 10.3390/md17010009

**Published:** 2018-12-24

**Authors:** Enoch Osei, Samuel Kwain, Gilbert Tetevi Mawuli, Abraham Kwabena Anang, Kofi Baffour-Awuah Owusu, Mustafa Camas, Anil Sazak Camas, Mitsuko Ohashi, Cristina-Nicoleta Alexandru-Crivac, Hai Deng, Marcel Jaspars, Kwaku Kyeremeh

**Affiliations:** 1 Marine and Plant Research Laboratory of Ghana, Department of Chemistry, School of Physical and Mathematical Sciences, University of Ghana, P.O. Box LG 56, Legon-Accra, Ghana; kofiosei0591@gmail.com (E.O.); kwainsamuel75@gmail.com (S.K.); gilberttet@gmail.com (G.T.M.); 2 Department of Parasitology, Noguchi Memorial Institute for Medical Research, University of Ghana, P.O. Box LG 581, Legon-Accra, Ghana; aanang@noguchi.ug.edu.gh (A.K.A.); kbaowusu@gmail.com (K.B.-A.O.); 3 Department of Bioengineering, Munzur University, 62000 Tunceli, Turkey; mustafacamas@gmail.com (M.C.); anilsazak@gmail.com (A.S.C.); 4 Section of Environmental Parasitology, Tokyo Medical and Dental University, Tokyo 113-8510, Japan; mikkvip@tmd.ac.jp; 5 Marine Biodiscovery Centre, Department of Chemistry, University of Aberdeen, Old Aberdeen, AB24 3UE Scotland, UK; r01cna14@abdn.ac.uk (C.-N.A.-C.); h.deng@abdn.ac.uk (H.D.); m.jaspars@abdn.ac.uk (M.J.)

**Keywords:** paenidigyamycin, plasmodium, trypanosome, leishmania, trichomonas, schistosome

## Abstract

A new alkaloid paenidigyamycin A (**1**) was obtained from the novel Ghanaian *Paenibacillus* sp. isolated from the mangrove rhizosphere soils of the *Pterocarpus santalinoides* tree growing in the wetlands of the Digya National Park, Ghana. Compound **1** was isolated on HPLC at t_R_ = 37.0 min and its structure determined by MS, 1D, and 2D-NMR data. When tested against *L. major*, **1** (IC_50_ 0.75 µM) was just as effective as amphotericin B (IC_50_ 0.31 µM). Against *L. donovani*, **1** (IC_50_ 7.02 µM) was twenty-two times less active than amphotericin B (IC_50_ 0.32 µM), reinforcing the unique effectiveness of **1** against *L. major*. For *T. brucei brucei*, **1** (IC_50_ 0.78 µM) was ten times more active than the laboratory standard *Coptis japonica* (IC_50_ 8.20 µM). The IC_50_ of 9.08 µM for **1** against *P. falciparum* 3d7 compared to artesunate (IC_50_ 36 nM) was not strong, but this result suggests the possibility of using the paenidigyamycin scaffold for the development of potent antimalarial drugs. Against cercariae, **1** showed high anticercaricidal activity compared to artesunate. The minimal lethal concentration (MLC) and minimal effective concentration (MEC) of the compound were 25 and 6.25 µM, respectively, while artesunate was needed in higher quantities to produce such results. However, **1** (IC_50_ > 100 µM) was not active against *T. mobilensis*.

## 1. Introduction

Sub-Sahara Africa (SSA) is burdened with a high incidence of parasitic infections, including schistosomiasis, trypanosomiasis, trichomoniasis, and leishmaniasis [1,2,3]. Currently, there is a rapid widespread development of resistance to prescription drugs for these parasitic neglected tropical diseases (pNTDs) [4,5,6,7,8]. The available number of drugs for treatment is exceptionally low and each of these has been under prescription for periods of no less than 30 years [9]. A rough estimate of the average of the prescription periods from time of discovery to-date for the top 10 antiparasitics, including praziquantel or oxamniquine, amphotericine, pentavalent antimonials, paromomycin, miltefosine, pentamidine, fluconazole or itraconazole, melarsoprol, eflornithine, and nifurtimox that are currently prescribed in the clinic is about 56 years. Furthermore, pNTD drugs only treat specific stages of parasite development, thereby complicating the treatment routines for patients and preventing complete parasite removal from the body [10,11]. Due to their long time prevalence in SSA, pNTDs have parasites and vectors whose genomes and lifecycles have evolved to assure their resilience and persistence [4]. Vectors responsible for pNTD transmissions are difficult to control and there is a huge debate concerning attempts to completely annihilate them [12]. Clearly, there is an urgent need to discover new scaffolds that would provide a platform for the development of future antiparasitics against pNTDs [13]. Microbial natural products provide the largest chemical and biological diversity in any drug discovery screening program compared to other natural sources, like plants and invertebrates, with the resupply problems mostly overcome with large-scale fermentation and heterologous expression [9,14,15,16]. Extreme un- or underexplored environments in SSA harbor novel microbes which, through millions of years of evolution, have evolved genomes that express chemically diverse molecules that could act as future drug leads for pNTDs and other diseases [17,18].

In the last four years, we have collected several soils and sediments from unexplored unique environments in the Ghanaian Western, Volta, and Brong Ahafo Regional wetlands. From these samples, we have isolated many cultivable strains of microbes, most of which give extracts which show antiparasitic activity.

**Figure 1:**
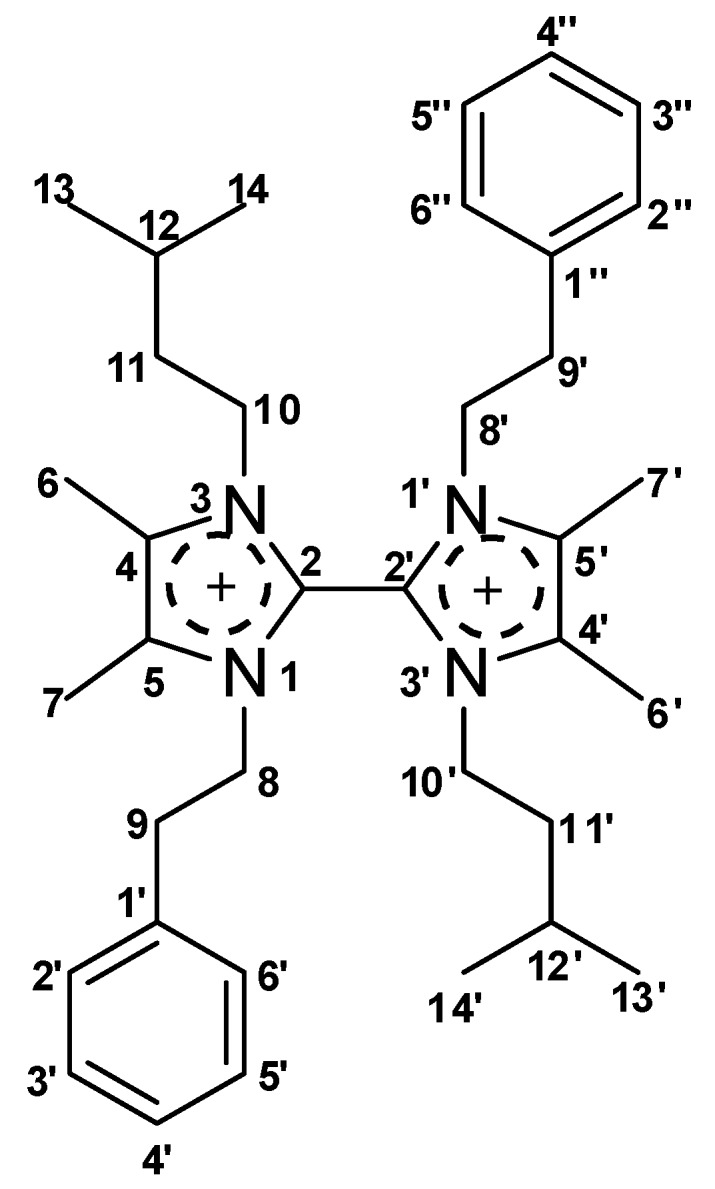
Structure of the imidazole-based alkaloid paenidigyamycin A (**1**) isolated from *Paenibacillus* sp. DE2SH.

Herein, we report on the isolation and purification of the Ghanaian *Paenibacillus* sp. strain DE2SH (GenBank Accession Number: MH091697) from mangrove rhizosphere soils collected from the Digya National Park in the Brong Ahafo Regional wetlands of Ghana. Culture of this strain at 28 °C, 220 rpm, in ISP2 media at pH 5.5 produced a crude extract which possessed antiparasitic activity against trypanosomes, schistosomes, leishmania, and plasmodium. Chemical profiling of this extract using high resolution electrospray ionization liquid chromatography mass spectrometry (HRESI-LC-MS) and nuclear magnetic resonance spectroscopy (NMR) showed the presence of an alkaloid which we have named paenidigyamycin (Figure 1). Solvent partitioning, Sephadex LH-20 size exclusion chromatography, and HPLC yielded the potent antiparasitic, paenidigyamycin A (**1**) (1.6 mg/L). The structure of this compound was determined using a combination of 1D- and 2D-NMR techniques with HRESI-LC-MS data.

## 2. Results and Discussion

### 2.1. Sediment Sample Collection Sites

The Ghanaian *Paenibacillus* sp. strain DE2SH (Supplementary Figure S1) was isolated from the mangrove rhizosphere soils of the *Pterocarpus santalinoides* tree growing in the wetland areas of the Digya National Park in the Brong Ahafo Region of Ghana (coordinates: 7°12′17.46″N and 0°05′35.01″E). This area has several patches of mangroves which are associated with the biggest reservoir man-made lake, Lake Volta. Water from the tributaries of the Volta ensures that the area occupied by this tree is covered with water throughout the year. Lake Volta is an open lake that is connected to the sea at the Ada Foah Basin, which in itself is a very dramatic site. The lake water is appreciably saline because, at high tide, seawater is pumped into the lake. Therefore, the diversity of micro- and macro-flora and fauna species is very high compared to lakes that have no connections with the sea. Several organisms that are meant to be entirely marine can be found in the lake and that includes marine microbes and fish [19]. There are a lot of species mixing in the mangroves associated with the lake, all the way to the Digya National Park.

### 2.2. Taxonomy of Strain DE2SH

**Figure 2:**
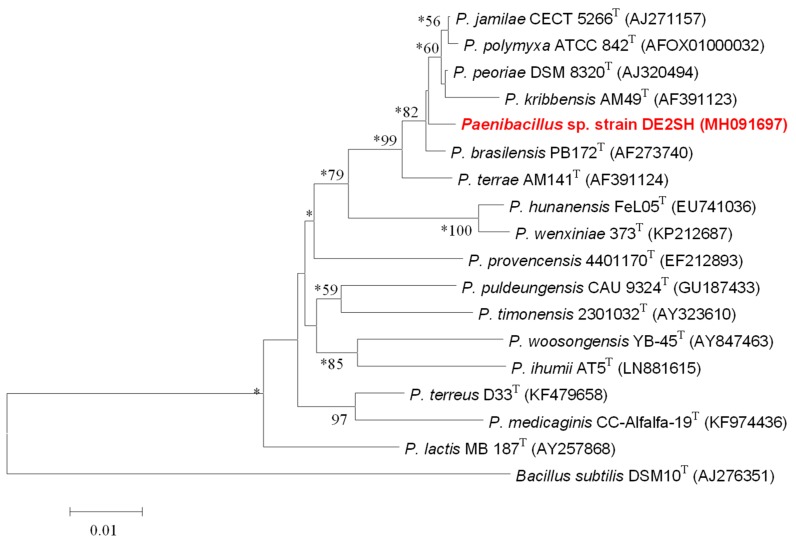
Neighbour-joining phylogenetic tree based on 16S rRNA gene sequences showing the relationships between strain DE2SH and other representative members of species of the genus *Paenibacillus*. *Bacillus subtilis* AJ276351T was used as an out-group. Asterisks (*) indicate branches that were also found using the maximum-parsimony [28] and maximum-likelihood methods [26]. Numbers at branch points indicate bootstrap percentages (based on 1000 replicates); only values >50 % are indicated. Bar, 0.01 substitutions per nucleotide position.

Identification of phylogenetic neighbors and calculation of pairwise 16S rRNA gene sequence similarities were achieved using the EzTaxon-e server [20]. Phylogenetic analysis was performed with the software package MEGA v.7.0 [21] after multiple sequence alignments with CLUSTAL W [22]. Distances were calculated according to the methods of Jukes-Cantor [23]. Phylogenetic trees were inferred using the neighbour-joining [24], maximum-parsimony [25], and maximum-likelihood [26] methods. The resultant tree topologies were evaluated by bootstrap analyses [27] based on 1000 re-samplings of the datasets. The comparative analysis of the 16S rRNA gene sequence of strain DE2SH revealed that it was phylogenetically affiliated to the genus *Paenibacillus* (Figure 2). *Paenibacillus* sp. strain DE2SH had the highest 16S rRNA gene sequence similarities with the type strains *P. jamilae* (99.11%), *P. polymyxa* (98.85%), *P. peoriaea* (98.84%), and *P. brasilensis* (98.70%), and other species of the genus *Paenibacillus* (<98.10%).

### 2.3. Structure Determination of Paenidigyamycin A (1)

**Figure 3:**
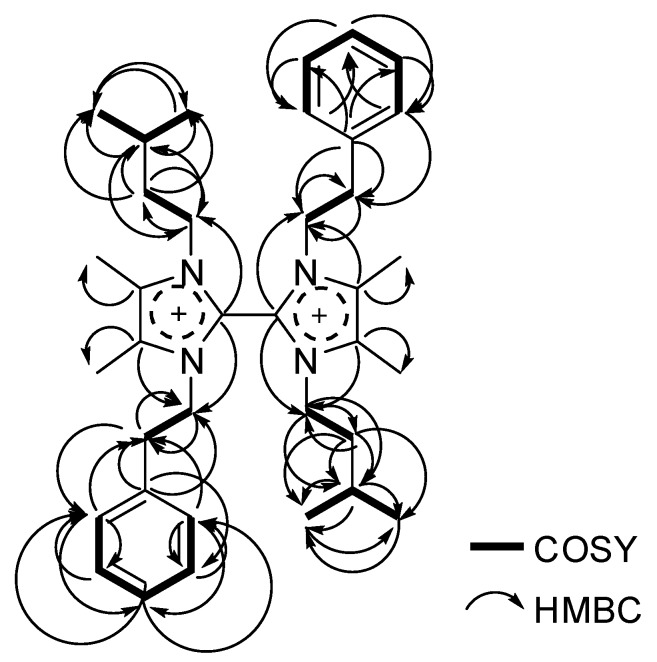
Key COSY (bold lines) and ^13^C-^1^H HMBC (arrows) correlations for compound **1**.

**Figure 4:**
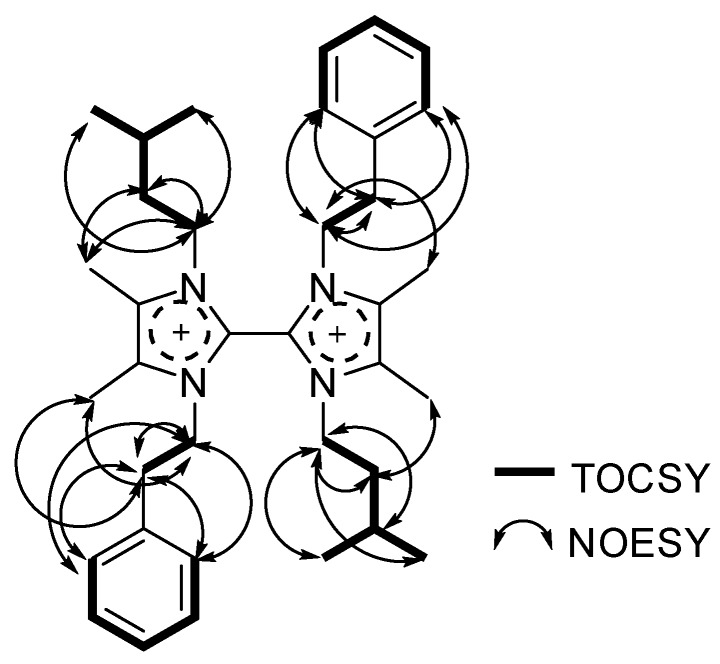
Key TOCSY (bold lines) and ROESY (double arrow) correlations for compound **1**.

Compound **1** was obtained at t_R_ of 37.0 min on semi-preparative reverse phase HPLC. It is a colorless and odorless oil when completely free of solvent. The HRESI-LC-MS of compound **1** gave an *m/z* of 271.2162, corresponding to a molecular formula of C_18_H_27_N_2_ (Δ = 0.001 and 7 degrees of unsaturation). It appears that compound **1** easily fragments in the mass spectrometer to yield two fragments with the same *m/z* of 271.2162 (Supplementary Figures S2–S4). Therefore, the *m/z* of **1** was calculated as 540.4186, which corresponds to the molecular formula of C_36_H_52_N_4_ with 14 degrees of unsaturation. Analysis of the ^1^H, ^13^C, and multiplicity edited gHSQCAD spectra of **1**, suggested the presence of 8 quaternary, 12 methine, 8 methylene, and 8 methyl carbons. The ^1^H-NMR chemical shifts δ_H_ 7.10 (4H, m, H-2′, H-2″, H-6′, H-6″), 7.31 (4H, m, H-3′, H-3″, H-5′, H-5″), and 7.29 (2H, m, H-4′, H-4″) provided direct evidence for the presence of two mono-substituted benzene rings. Mono-substituted benzene rings have a well-known reputation of producing weakly resolved multiplicity patterns due to the identical coupling constants which exist in such systems. Detailed analysis of the gCOSY spectrum showed correlations H-2′/H-3′, H-2″/H-3″, H-5′/H-6′, and H-5″/H-6″, which were further supported by similar 2D-TOCSY correlations, including H-2′/H-4′, H-6′/H-4′, H-2″/H-4″, and H-6″/H-4″ to fully establish these aromatic spin systems. Important gHMBCAD correlations, such as C-1′ to H-3′, C-1′ to H-5′, C-1″ to H-3″, and C-1″ to H-5″, confirmed that the only quaternary carbon outlet within the aromatic systems was δ_C_ 137.8 (C-1′ and C-1″). Most importantly, gHMBCAD correlations C-1′ to H-8, C-1″ to H-8′, C-1′ to H-9, and C-1″ to H-9′ showed that the two aromatic systems were both attached to an ethyl group with carbon atoms at δ_C_ 49.7 (C-8), 36.7 (C-9), 49.2 (C-8′), and 37.1 (C-9′), and δ_H_ 4.40 (2H, t, *J* = 6.8 Hz, H-8), 3.12 (2H, t, *J* = 6.7 Hz, H-9), 4.33 (2H, t, *J* = 6.9 Hz, H-8′), and 3.04 (2H, t, *J* = 6.9 Hz, H-9′), respectively. The gCOSY correlations H-8/H-9 and H-8′/H-9′ confirmed these two isolated spin systems with the lower methylene chemical shift carbons δ_C_ 36.7 (C-9) and 37.1 (C-9′) attached to the mono-substituted benzene rings, while the higher methylene chemical shift carbons δ_C_ 49.7 (C-8) and 49.2 (C-8′) were attached to the nitrogen of possible heteroaromatic systems. Furthermore, two isopentyl spin systems were identified with δ_H_ 4.03 (2H, m, H-10), 1.57 (2H, m, H-11), 1.48 (1H, n, *J* = 6.8 Hz, H-12), 0.96 (6H, d, *J* = 6.6 Hz, H-13, H-14) and 4.13 (2H, m, H-10′), 1.73 (2H, m, H-11′), 1.67 (1H, n, *J* = 6.8 Hz, H-12′), and 1.02 (6H, d, *J* = 6.5 Hz, H-13′, H-14′). The δ_H_ 4.03 (2H, m, H-10) and 4.13 (2H, m, H-10′) with δ_C_ 46.4 (C-10) and 46.6 (C-10′) were obviously connected to the nitrogen of some heteroaromatic systems. With two ethyl benzenes and two isopentyl substructures completely solved, the presence of the imidazole moiety was deduced by the subtraction of the known molecular formulas from the overall formula and the subsequent calculation of unsaturation numbers. The presence of the two imidazole rings was further confirmed by the rather low δ_C_ 8.1 (C-6), 8.1 (C-7) and δ_C_ 8.2 (C-6′), 8.0 (C-7′), which is very characteristic of methyl carbons attached to imidazole. The six imidazole δ_C_ 135.4 (C-2), 135.5 (C-2′), 128.4 (C-4), 128.5 (C-4′), 128.4 (C-5), and 128.5 (C-5′) were identified with gHMBCAD correlations C-4 to H-6, H-10, C-5 to H-7, H-8 and C-4′ to H-6′, H-10′, C-5′ to H-7′, and H-8′, confirming the positions of the methyl substituents on the imidazole ring, as well as the pattern of substitution of the isopentyl and ethyl benzene moieties on the nitrogen of the imidazole rings. The structure of paenidigyamycin A was further confirmed by the analysis of NOESY data, which along with the other 2D-NMR data, are summarized in Figure 3 and Figure 4 and Table 1. The full details of all the NMR data with correlations can be found in the Supplementary Table S1a,b, but the summary is shown in Table 1.

### 2.4. Antiparasitic and Antibacterial Activity of Paenidigyamycin A

Compound **1** did not show any positive response when tested against a range of cancer cell lines in our laboratory. These cancer cell lines include: Jurkat; Human T-lymphoblastic leukemia and HL-60; Human promyelocytic leukemia cells, which are suspension cells; followed by PC-3; Human Prostate Cancer, HepG2; Human hepatocellular carcinoma, LNCap; Human prostate cancer, and MCF-7; Human breast cancer, which are adhesive cells. The antibacterial activity of **1** ranged from moderate to weak when the compound was tested against *Escherichia coli*, *Staphylococcus aureus*, *Bacillus cereus*, *Shigella flexneri*, *Salmonella paratyphi* B, and *Listeria monocytogenes* (Table 2). However, against *Plasmodium falciparum* 3d7 strain, *Trypanosoma brucei brucei*, *Leishmani donovani*, and *Leishmania major*, **1** consistently produced very potent results (Table 3). Against *L. major*, compound **1** (IC_50_ 0.75 µM) was just as effective as the rather expensive and structurally complicated amphotericin B (IC_50_ 0.31 µM). Given the simplicity in the structure of **1**, synthetic production of this compound could easily be commercialized. Against *L. donovani*, compound **1** (IC_50_ 7.02 µM) was twenty-two times less active than amphotericin B (IC_50_ 0.32 µM), but this result is interesting because it reinforces the unique effectiveness of **1** against *L. major*. For *T. brucei brucei*, **1** (IC_50_ 0.78 µM) was ten times more active than the laboratory standard *Coptis japonica* (IC_50_ 8.20 µM), which is reported to contain the alkaloid berberine (Table 3). The IC_50_ of 9.08 µM for **1** against *P. falciparum* 3d7 strain compared to artesunate (IC_50_ 36 nM) is not strong, but this result suggests the possibility of using the paenidigyamycin scaffold for the development of potent antiparasitic drugs.

Compound **1** showed varying anticercaricidal potency against *Schistosoma mansoni* cercariae, with pronounced activity at elevated concentrations of the compound. The effect of incubation with different concentrations of the compound on the viability of cercariae for up to 180 min is shown in Supplementary Figure S5. The exposure of *S. mansoni* cercariae to compound **1** for 30, 60, 90, 120, 150, and 180 min showed a steady increase in the mortality rates of the parasite. In the absence of the compound, cercariae showed normal viability, without any morphological changes (tail loss) for up to 180 min. However, after incubation with the compound at the highest concentration of 100 μM, cercariae viability decreased significantly within 30 min, with complete mortality at 60 min. At concentrations of 25–100 μM, cercariae viability decreased significantly within 60 min, with no movement observed. At 90 min, cercariae incubated with 12.5 μM showed slowed movements and the same was observed for 6.25 μM after 120 min. After 150 min and incubation with 25, 50, and 100 μM concentrations of the compound, there was 100% mortality, with changes in viability and morphology for 6.25 and 12.5 μM. The MLC and MEC of compound **1** on cercariae after 180 min were 25 μM and 6.25 μM, respectively. The standard artesunate which was chosen for these experiments as a result of its reported effectiveness against the juvenile forms of the schistosome worms was required in higher concentrations to produce the same effects as compound **1** [29]. The LC_50_ for the different time points for artesunate were calculated at 60 min (33.7 μM), 90 min (25.0 μM), 120 min (20.2 μM), 150 min (16.44 μM), and 180 min (15.08 μM). These results suggest that the paenidigyamycin scaffold could provide possible future antiparasitic drugs and it is our intention to investigate this compound further. We hope to synthesize the compound and derivatives in order to determine and possibly compare the antiparasitic activities.

However, when tested against *Trichomonas mobilensis*, compound **1** did not give any good results (Table 4).

### 2.5. Possible Biosynthesis of Paenidigyamycin A (**1**)

**Scheme 1:**
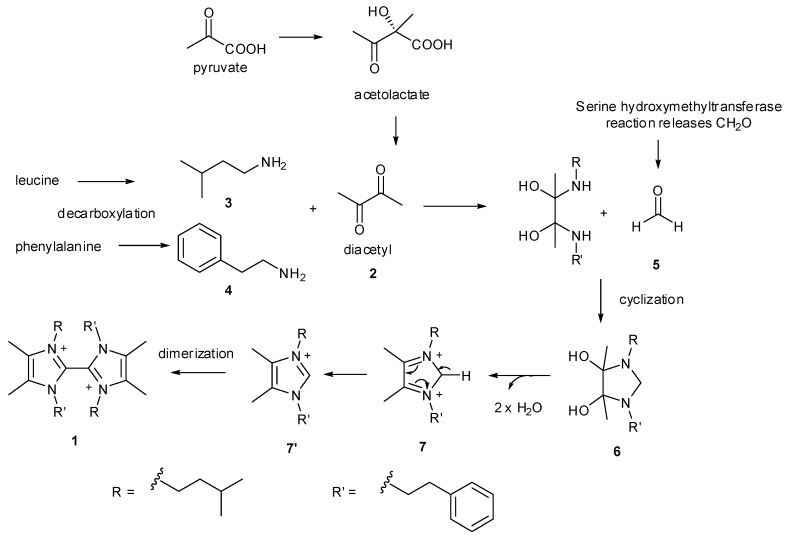
A Proposed Model for the Biosynthesis of Paenidigyamycin A (**1**).

Detailed inspection of the structure of paenidigyamycin A (**1**) led to the speculation that **1** could be biosynthesized through the condensation of four units: diacetyl (**2**), 3-methylbutanamine (**3**), 2-phenyl-ethanamine (**4**), and formaldehyde (**5**). Diacetyl (**2**) is most likely derived from acetolactate through non-enzymatic oxidative decarboxylation. The precursors **3** and **4** are also the decarboxylation products of the corresponding amino acids leucine and phenylalanine, respectively. The cyclization intermediate **6** will then undergo dehydration to yield **7**, followed by tautomerization to yield **7′**. Finally, the dimerization occurs to provide **1**, probably through a radical reaction between the two monomers (Scheme 1).

## 3. Experimental Section

### 3.1. General Experimental Procedures

1D and 2D NMR data were recorded on a Bruker AVANCE III HD Prodigy (BRUKER, Sylvenstein, Germany) at 500 and 125 MHz for ^1^H and ^13^C, respectively. This instrument was optimized for ^1^H observation with pulsing/decoupling of ^13^C and ^15^N, with 2H lock channels equipped with shielded z-gradients and cooled preamplifiers for ^1^H and ^13^C. The ^1^H and ^13^C chemical shifts were referenced to the solvent signals (δ_H_ 3.31 and δ_C_ 49.00 ppm in CD_3_OD). High-resolution mass spectrometry data were measured using a ThermoScientific LTQXL-Discovery Orbitrap (Thermo Scientific, Bremen, Germany) coupled to an Accela UPLC-DAD system. The following conditions were used for mass spectrometric analysis: capillary voltage 45 V, capillary temperature 320 °C, auxiliary gas flow rate 10−20 arbitrary units, sheath gas flow rate 40−50 arbitrary units, spray voltage 4.5 kV, and mass range 100−2000 amu (maximum resolution 30,000). Semi-preparative HPLC purifications were carried out using a Phenomenex Luna reverse-phase (C18 250 × 10 mm, L × i.d.) column connected to a Waters 1525 Binary HPLC pump Chromatograph with a 2998 photodiode array detector (PDA), column heater, and in-line degasser. Detection was achieved on-line through a scan of wavelengths from 200 to 400 nm. This system was also used to record the UV profile for the compounds. IR was measured using a PerkinElmer FT-IR (UATR Two) spectrometer. All solvents were HPLC grade. Sephadex LH-20 and HP-20 resin were obtained from Sigma Aldrich (Munich, Germany).

### 3.2. Identification of Strain DE2SH

Sanger and whole-genome sequencing of the strain was carried out by the DNA Sequencing Facility, Department of Biochemistry, University of Cambridge. In this work, genomes were assembled into a small number of high quality scaffolds by Dr. Markiyan Samborskyy using a combination of shotgun and long-range mate pair Illumina sequencing data. A nearly complete sequence (1476 bp) of the 16S rRNA gene obtained in the present study was used for the identification of isolated strain DE2SH and for the construction of a phylogenetic tree. The 16S rRNA gene sequence was deposited in the GenBank database (GenBank accession number: MH091697).

### 3.3. Fermentation

An Autoclaved Erlenmeyer flask (250 mL) plugged with non-absorbent cotton wool containing 50 mL of ISP2 (10 g of malt extract, 4 g each of yeast extract and D-glucose) fermentation media in distilled water with pH 5.5 was directly inoculated with spores of strain DE2SH and incubated at 28 °C at 220 rpm for three days. This seed culture was subsequently used to inoculate nine autoclaved 1 L Erlenmeyer flasks, each containing 200 mL ISP2 media at pH 5.5 and plugged with non-absorbent cotton wool. The 1 L flasks were incubated at 28 °C at 220 rpm for 21 days. Two days before the culture incubation period was complete, autoclaved HP-20 resin was added at 50 g/L to each of the flasks under sterile conditions and the flasks were returned to the incubator.

### 3.4. Extraction and Purification

The *Paenibacillus* sp. strain DE2SH fermentation broth (1.8 L) was filtered through a piece of glass wool under suction in a Buchner funnel to separate the supernatant from the mycelia. The supernatant was extracted with EtOAc and the mycelia and HP-20 resin were placed in a 1 L flask and extracted sequentially and alternatively with MeOH and CH_2_Cl_2_. All extracts were combined and evaporated under reduced pressure to obtain a total crude extract (2.84 g). The total crude extract was subjected to a modification of Kupchan’s solvent partitioning process [30] that gave the four fractions FH (0.65 g), FD (0.48 g), FM (0.32 g), and WB (1.08 g), with the compounds of interests concentrated in the polar FM and WB fractions. The FM fraction was then subjected to Sephadex LH-20 size exclusion chromatography by gravity to obtain six fractions that were labelled FM-SFA-F. Phytochemical screening with ninhydrin on TLC plates followed by ^1^H-NMR showed the compound of interest to be concentrated in the FM-SFD (0.15 g). Fraction FM-SFD was therefore subjected to semi-preparative HPLC separation and purification using a Phenomenex Luna C18 column (C18 250 × 10 mm, L × i.d.). Gradients of Solvent A: H_2_O (0.1% HCOOH) and Solvent B: CH_3_CN (0.1% HCOOH) (100% A to 100% B in 30 min and hold for 30 min) were used as eluents with column flow rates set at 1.5 mL/min to afford compound **1** (1.2 mg, t_R_ = 37.0 min).

Paenidigyamycin A (**1**): colorless and odorless substance; IR (neat) ν_max_ 3088, 3064, 3028, 2962, 2930, 2872, 1661, 1516, 1496, 1374, 751, 703 cm^−1^; UV (H_2_O:CH_3_CN) λ_max_ 210, 254, 280 nm; for ^1^H and ^13^C NMR data, see Table 1; Mass spectrometry data is detailed in Supplementary Figures S2–S4.

### 3.5. Bioassay Reagents

Fetal Bovine Serum (FBS), Roswell Park Memorial Institute (St. Louis, MO, USA); (RPMI) 1640, IMDM, M-199, 2-[4-(2-hydroxyethyl)piperazin-1-yl]ethanesulfonic acid (HEPES), YI-S, Adult Bovine Serum (ABS), Gentamycin, Penicillin-Streptomycin-L-Glutamine (PSG), Artesunate, Alamar dye, Dimethyl sulfoxide (DMSO), Sodium citrate, Adenine, Sodium bicarbonate (NaHCO_3_), AlbuMax II, Sodium chloride (NaCl), Potassium chloride (KCl), Sodium Phosphate Dibasic (Na_2_HPO_4_), Sodium Phosphate Monobasic (KH_2_PO4), and Sodium hydroxide (NaOH), were purchased from Sigma-Aldrich, USA. All other chemicals and reagents were of analytical grade.

### 3.6. Compound Preparation for Bioassay

A stock solution of compound **1** was prepared at a concentration of 100 mM. This was achieved by drying the compound with nitrogen gas and weighing on a balance (AND GH-120, A and D Company Limited, Tokyo, JAPAN) to ascertain the mass of compound. Compound **1** was dissolved in an appropriate amount of DMSO to attain the desired concentration. The stock solution was vortexed (MSI Minishaker. IKA Company, Osaka, JAPAN) and filter sterilized into a vial through 0.45 µm millipore filters under sterile conditions and stored at −20 °C until use.

### 3.7. Cell Culture

Erythrocytes were obtained from the blood of consenting volunteers (Blood group O+). Venous blood was drawn into containers with citrate phosphate dextrose (CPD) solution and kept at 4 °C overnight. This preparation was centrifuged at 2000 rpm for 10 min to separate the serum and buffy coat (KUBOTA 5200 Centrifuge, Kubota Corporation, Osaka, JAPAN). Packed erythrocytes were washed three times with parasite washing medium (RPMI 1640, buffered with, 50 µg/mL gentamicin and 2 mM L-glutamine). Each washing step was carried out by the addition of wash medium, pipetting up and down thrice, centrifuging at 2000 rpm for 10 min, and discarding the suspended medium. After washing, wash medium was added to the packed erythrocytes and stored at 4 °C until ready to use. Washed red blood cells were stored and used for up to two weeks maximum, after which new blood was collected. Erythrocytic stages of the malaria parasite (*Plasmodium falciparum*-chloroquine sensitive strain 3D7) were cultured in 25 mL flasks using the method of Trager and Jensen (1976), with modifications. Erythrocytes were maintained at 2% haematocrit (*v*/*v*) cell suspension in complete malaria parasite medium (RPMI 1640, buffered with 25 mM HEPES, supplemented with 7.5% NaHCO_3_, 25 ug/mL gentamycin, 5% heat-inactivated human O+ serum; from consenting subjects) and 5 mg/mL AlbuMax II, and incubated at 37 °C under gas conditions of 2% O_2_, 5% CO_2_, and 93% N_2_. Parasite growth and development were monitored with a Giemsa stained thin blood smear. Parasite culture was purified by using 5% sorbitol to obtain matured erythrocyte parasitic stages (late trophozoites and schizonts) from uninfected cells. The matured erythrocyte parasitic stages (purity > 90%) obtained were used to screen the compound for antimalarial activity.

The GUTat 3.1 strain of the bloodstream form of *T. brucei* parasites was used in this study. Parasites were cultured *in vitro* according to the conditions established previously by Yabu et al. in 1998 [31]. Parasites were used when they reached a confluent concentration of 1 × 10^6^ parasites/mL. Estimation of parasitemia was done with the Neubauer counting chamber. Parasites were diluted to a concentration of 3 × 10^5^ parasites/mL with IMDM medium and used for the drug assay.

The log-phase promastigotes of *L. donovani* (D10) and *L. major* (NR48815) were cultured in M-119 growth medium with a working concentration of 6 × 10^6^ cells/mL. Parasites were used when they reached a confluent concentration of 1 × 10^6^ parasite/mL. Estimation of parasitemia was done with the Neubauer counting chamber. Parasites were diluted to a concentration of 3 × 10^5^ parasites/mL with M199 medium and used for the drug assay.

### 3.8. Preparation of Cercarial Suspension

Schistosome cercariae were obtained from experimentally infected *Biomphalaria pfeifferi* snails. Briefly, five laboratory four week-infected snails known to be shedding cercariae were placed in a test tube containing 2 mL of distilled water and allowed to shed cercariae by exposing them to artificial light at 28 ± 1 °C for 2 h. The number of cercariae in 50 μL was counted microscopically in triplicate samples and the average count used for anticercariae evaluation was 20 cercariae per well.

### 3.9. Bio-Assays

#### 3.9.1. Screening for Anti-Malaria Activity Using the SYBR Green I Assay

Screening for antimalarial activity was achieved using the SYBR Green I fluorescence assay as described by Smilktein et al. in 2004 [32,33], with some modifications. Serial dilutions of the standard (artesunate) which served as an experimental control and stock solutions of compounds to yield final concentrations ranging from 15.63 nM to 500 nM and 1.95 µg/mL to 250 µg/mL, respectively, were prepared. The matured erythrocyte parasitic stages were treated with the compound and washed erythrocytes in 96-well plates and incubated with complete malaria parasite medium until harvested after 48 h. Slides were prepared and percent parasitaemia was determined by counting the number of infected cells in a total of 500 erythrocytes, in the Giemsa-stained thin blood smear. Briefly, an aliquot of 5 µL per concentration of the standard drug and compound was dispensed into test wells. About 95 µL of complete malaria parasite medium with washed erythrocytes at 2% haematocrit and the purified matured erythrocyte parasitic stages (1% parasitaemia) was added, and incubated at 37 °C under gaseous conditions as stated above, while untreated erythrocytes were used as the control. Wells containing erythrocytes at 2% haematocrit, infected erythrocytes at 2% haematocrit, and complete parasite medium alone served as negative controls, positive controls, and blank controls, respectively. Furthermore, wells containing infected parasites and 0.1% DMSO served as reference controls. The final volume per well was 100 µL. Plates were then incubated for 24 h as described above in the cultivation of malaria parasites. About a 100 µL aliquot of 2.5× buffered SYBR Green I (0.25 µL of SYBR Green I/mL of phosphate buffer saline) was added to each well after incubation.

#### 3.9.2. *In Vitro* Viability Test for Trypanosome Parasites

The Alamar Blue assay was carried out on treated and untreated trypanosome parasites to ascertain their viability. The assay was performed in a 96-well plate following the manufacturer’s instructions, with modification. Briefly, 1.5 × 10^4^ parasites were seeded with varied concentrations of the compound ranging from 0 µM to 100 µM. Final concentrations of DMSO were kept at 0.1%. After the incubation of parasites with or without the compound for 24 h at 37 °C in 5% CO_2_, 10% Alamar Blue dye was added, and the parasites were incubated another 24 h in darkness. After a total of 48 h, the plate was read for absorbance at 540 nm using the Tecan Sunrise Wako spectrophotometer, AUSTRIA GmbH. The trend curve was drawn to obtain a 50% inhibitory concentration (IC_50_) for the compound.

#### 3.9.3. *In Vitro* Viability Test for Leishmania Parasites

The Alamar Blue assay was carried out on treated and untreated leishmania parasites to ascertain their viability. The assay was performed in a 96-well plate following the manufacturer’s instructions, with modification. About 3 × 10^5^ parasites were seeded with varied concentrations of the compound ranging from 0 µM to 100 µM. Final concentrations of DMSO were kept at 0.1%. After incubation of parasites with or without the compound for 24 h at 28 °C, 10% Alamar Blue dye was added, and the parasites were incubated for another 24 h in darkness. After a total of 48 h, the plate was read for absorbance at 540 nm using the Tecan Sunrise Wako spectrophotometer, AUSTRIA GmbH (Salzburg, Austria). The trend curve was drawn to obtain a 50% inhibitory concentration (IC_50_) for the compound.

#### 3.9.4. *In Vitro* Cercariacidal Activity Test

Varied concentrations of the compound ranging from 0 µM to 100 µM were freshly prepared in a 24-microtiter well plate (Costar) and analyzed alongside the positive control (artesunate 10 μM). An average number of 20 freshly shed cercariae were transferred into each well plate (Costar) with a micropipette. The same number of cercariae were placed in a well containing 0.1% DMSO as the negative control. All experiments were carried out in duplicate and repeated. Mobility and viability of the Schistosoma infectious stage (cercariae) were observed for 2 h 30 min at 30 min intervals since infectivity of cercariae is known to be rapidly lost after 12 h [34,35]. Unaffected free swimming larvae, immobile, and dead cercariae at the bottom of the wells were observed at 4× magnification with an inverted microscope (Olympus CK 300, OLYMPUS, Tokyo, Japan). Survival and mortality at a successive interval of 15, 30, 60, 90, 120, and 150 min were recorded. Cercariae were presumed dead when they stopped moving and sank down to the bottom of the well with their tails detached [35]. The LC_50_ values of the compound on schistosome cercariae were determined at 1 and 2 h. The minimal lethal concentration (MLC), which is the minimum concentration needed to kill all cercariae, and the minimal effective concentration (MEC), which is the minimum concentration needed to observe any change in viability or morphology of cercariae, were determined after 2 h.

#### 3.9.5. *In-Vitro* Susceptibility Testing of Trichomonas Mobilensis

Compound **1** was prepared at a concentration of 0–100 µM in 96-well plates. Trophozoites of *Trichomonas mobilensis* were seeded in the well plates at a concentration of 2 × 10^6^ cells/mL. The plates were sealed with seal stickers to provide anaerobic conditions for parasites and transferred into an Anaero Pack jar with gas generators. The jar was incubated at 28 °C for 48 h. Parasites were transferred into black well plates and 100 µL ATP bioluminescent was added before incubating for 20 min, followed by luminescence readings using the GloMAX multidetection system (Promega Corp., Madison, WI, USA).

## 4. Conclusions

The imidazole ring is an important, very common functionality present in many potent bioactive natural and synthetic molecules and drugs. When an imidazole moiety is introduced into a compound, it modulates the physicochemical properties of the compound. The physical and chemical properties of the imidazole ring make it well-suited for weak to strong binding to a variety of enzymes and receptors, resulting in a broad spectrum of activities. Consequently, imidazole-based compounds have been found to possess anticancer, antifungal, antibacterial, antitubercular, anti-inflammatory, antineuropathic, antihypertensive, antihistaminic, antiobesity, antiviral, and antiparasitic activities [36]. Megazol, benznidazole, and metronidazole are well-known examples of potent imidazole-based antiparasitics [36]. Countless bioactive microbial peptides like bacitracin and gassericin contain the imidazole moiety due to the incorporation of the amino acid histidine, but molecules like urocanic acid, leuhistin, and tricladins A and B show the potential for further imidazole metabolism by microbes. The interesting antiparasitic activity shown by paenidigyamycin A in this project is therefore not surprising and offers an opportunity for further development of this pharmacophore.

## Figures and Tables

**Table 1:** 1D and HMBC NMR Spectroscopic Data for Paenidigyamycin A (**1**) in CD_3_OD, in ppm.

#	δ_C_ mult	δ_H_ mult (*J* Hz)	HMBC	#	δ_C_ mult	δ_H_ mult (*J* Hz)	HMBC
1-N				1′-N			
2	135.4, C		8, 10	2′	135.5, C		8′, 10′
3-N				3′-N			
4	128.4, C		10, 6	4′	128.5, C		10′, 6′
5	128.4, C		8, 7	5′	128.5, C		8′, 7′
6	8.1, CH_3_	2.27, s		6′	8.2, CH_3_	2.31, s	
7	8.1, CH_3_	2.22, s		7′	8.0, CH_3_	2.06, s	
8	49.7, CH_2_	4.40, t (6.8)	9	8′	49.2, CH_2_	4.33, t (6.9)	9′
9	36.7, CH_2_	3.12, t (6.7)	8, 2′, 6′	9′	37.1, CH_2_	3.04, t (6.9)	8′
10	46.4, CH_2_	4.03, m	12, 11	10′	46.6, CH_2_	4.13, m	12′, 11′
11	39.5, CH_2_	1.57, m	13, 14, 10, 12	11′	39.5, CH_2_	1.73, m	13′, 14′, 12′, 10′
12	26.5, CH	1.48, n (6.8)	13, 14, 10	12′	26.9, CH	1.67, n (6.8)	13′, 14′, 11′, 10′
13	22.5, CH_3_	0.96, d (6.6)	14	13′	22.5, CH_3_	1.02, d (6.5)	14′
14	22.5, CH_3_	0.96, d (6.6)	13	14′	22.5, CH_3_	1.02, d (6.5)	13′
1′	137.8, C		9, 8, 3′, 5′	1″	137.8, C		3″, 5″, 9′, 8′
2′	130.0, CH	7.10, m	9, 4′, 3′, 5′	2″	130.0, CH	7.10, m	4″, 3″, 5″, 9′
3′	130.0, CH	7.31, m	2′, 6′	3″	130.0, CH	7.31, m	2″, 6″
4′	128.4, CH	7.29, m	2′, 6′	4″	128.4, CH	7.29, m	2″, 6″
5′	130.0, CH	7.31, m	2′, 6′	5″	130.0, CH	7.31, m	2″, 6″,
6′	130.0, CH	7.10, m	9, 4′, 3′, 5′	6″	130.0, CH	7.10, m	4″, 3″, 5″, 9′

**Table 2:** Antibacterial bioactivity data of compound **1** with ampicillin as the positive control.

Compound (1)	Microbe	IC_50_ (µM)	Positive Control	IC_50_ (µM)
Paenidigyamycin A	*E. coli*	76.98	Ampicillin	10.44
	*S. aureus*	11.87	Ampicillin	0.18
	*B. cereus*	97.23	Ampicillin	1.70
	*S. flexneri*	56.98	Ampicillin	1.76
	*S. paratyphi B*	57.98	Ampicillin	1.53
	*L. monocytogenes*	18.98	Ampicillin	2.71

**Table 3:** Antiparasitic bioactivity data for compound **1**.

Compound (1)	Parasite	IC_50_ (µM)	Positive Control	IC_50_
Paenidigyamycin A	*P. falciparum* 3d7	9.08	Artesunate	36 nm
	*T. brucei brucei*	0.78	*Coptis japonica*	8.20 µM
	*L. donovani*	7.02	Amphotericin B	0.32 µM
	*L. major*	0.75	Amphotericin B	0.31 µM

**Table 4:** In-vitro susceptibility results for *Trichomonas mobilensis.*

Compound (1)	Parasite	IC_50_ (µM)	Positive Control	IC_50_ (µM)
Paenidigyamycin A	*T. mobilensis*	> 100	Metronidazole	5.2

## Supplementary Materials

The following are available online at http://www.mdpi.com/1660-3397/17/1/9/s1, Raw 1D- and 2D-NMR spectra of compound **1** are provided in Figures S6–S12. Figure S13, Modified Kupchan solvent partitioning of the crude extract of *Paenibacillus* sp. DE2SH gives FH, FD, FM and WB fractions. Figure S14, Sephadex LH-20 Chromatography of FM fraction followed by Semi-preparative HPLC gives pure Paenidigyamycin A (**1**). Figure S15, Schematic representation of a feasible fragmentation pathway for possible Paenidigyamycin A (**1**) analogue under HRESI-LC-MS-MS conditions. Figure S16, HRESI-LC-MS shows the possible presence of a Paenidigyamycin A (**1**) analogue. Figure S17, HPLC Chromatogram of Paenidigyamycin A (**1**) with UV profile.
