# Supplementary material for: Paenidigyamycin A, Potent Antiparasitic Imidazole Alkaloid from the Ghanaian Paenibacillus sp. DE2SH
Source: Mar Drugs. 2018 Dec 24;17(1):9. doi: 10.3390/md17010009 (PMC6356793; doi:10.3390/md17010009)
Supplement: Supplementary file 1 [file marinedrugs-17-00009-s001.pdf]

marine drugs

Supplementary Material

Paenidigyamycin A, Potent Antiparasitic Imidazole Alkaloid from the Ghanaian *Paenibacillus* sp. DE2SH

Enoch Osei [1,†], Samuel Kwain [1,†], Gilbert Tetevi Mawuli [1], Abraham Kwabena Anang [2], Kofi Baffour-Awuah Owusu [2], Mustafa Camas [3], Anil Sazak Camas [3], Mitsuko Ohashi [4], Cristina-Nicoleta Alexandru-Crivac [5], Hai Deng [5], Marcel Jaspars [5] and Kwaku Kyeremeh [1,*]

[1] Marine and Plant Research Laboratory of Ghana, Department of Chemistry, School of Physical and Mathematical Sciences, University of Ghana, P.O. Box LG 56, Legon-Accra, Ghana; kofiosei0591@gmail.com (E.O.); kwainsamuel75@gmail.com (S.K.); gilberttet@gmail.com (G.T.M.)

[2] Department of Parasitology, Noguchi Memorial Institute for Medical Research, University of Ghana, P.O. Box LG 581, Legon-Accra, Ghana; aanang@noguchi.ug.edu.gh (A.K.A.); kbaowusu@gmail.com (K.B.A.O.)

[3] Department of Bioengineering, Munzur University, 62000 Tunceli, Turkey; mustafacamas@gmail.com (M.C.); anilsazak@gmail.com (A.S.C.)

[4] Section of Environmental Parasitology, Tokyo Medical and Dental University, Tokyo, Japan; mikkvip@tmd.ac.jp

[5] Marine Biodiscovery Centre, Department of Chemistry, University of Aberdeen, Old Aberdeen, AB24 3UE, Scotland, UK; r01cna14@abdn.ac.uk (C.N.A.C.); h.deng@abdn.ac.uk (H.D.); m.jaspars@abdn.ac.uk (M.J.)

† These authors contributed equally to this paper

* Correspondence: kkyeremeh@ug.edu.gh; Tel.: +233-20-789-1320

Contents

Figure S1. *Paenibacillus* sp. DE2SH growing on ISP2 pH 5.5 agar plate....................................3

Figure S2. Schematic representation of feasible fragmentation pathway for Paenidigyamycin A (**1**) under HRESI-LC-MS conditions..3

Figure S3. HRESI-LC-MS shows chromatogram that confirms the fragmentation pathway for Paenidigyamycin A (**1**). 4

Figure S4. HRESI-LC-MS for crude FM extracts of strain DE2SH. 4

Figure S5. Effect of different concentrations of compound **1** on the viability of *Schistosoma mansoni* cercariae. 5

Table S1a. Full 1D and 2D NMR Spectroscopic data for Paenidigyamycin A (**1**) in CD_3OD, in ppm. 5

Table S1b....6

Figure S6. ^{1}H NMR spectrum (500 MHz) of Paenidigyamycin A (**1**) in CD_3OD. 7

Figure S7. DEPT135° spectrum of Paenidigyamycin A (**1**) in CD_3OD. 8

Figure S8. HSQC spectrum (500 MHz) of Paenidigyamycin A (**1**) in CD_3OD. 9

Figure S9. COSY spectrum (500 MHz) of Paenidigyamycin A (**1**) in CD_3OD. 10

Figure S10. 2D-TOCSY spectrum (500 MHz) of Paenidigyamycin A (**1**) in CD_3OD. 11

Figure S11. HMBC spectrum (500 MHz) of Paenidigyamycin A (**1**) in CD_3OD. 12

Figure S12. NOESY spectrum (500 MHz) of Paenidigyamycin A (**1**) in CD_3OD. 13

Figure S13. Modified Kupchan solvent partitioning of the crude extract of *Paenibacillus* sp. DE2SH gives FH, FD, FM and WB fractions. 14

Figure S14. Sephadex LH-20 Chromatography of FM fraction followed by Semi-preparative HPLC gives pure Paenidigyamycin A (**1**). 15

Figure S15. Schematic representation of a feasible fragmentation pathway for possible Paenidigyamycin A (**1**) analogue under HRESI-LC-MS-MS conditions. 16

Figure S16. HRESI-LC-MS shows the possible presence of a Paenidigyamycin A (**1**) analogue...17

Figure S17. HPLC Chromatogram of Paenidigyamycin A (**1**) with UV profile.......................18

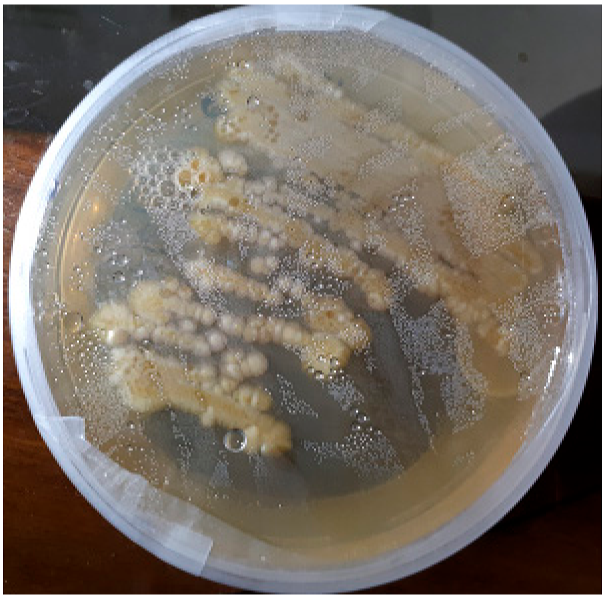

Figure S1. *Paenibacillus* sp. DE2SH growing on ISP2 pH 5.5 agar plate.

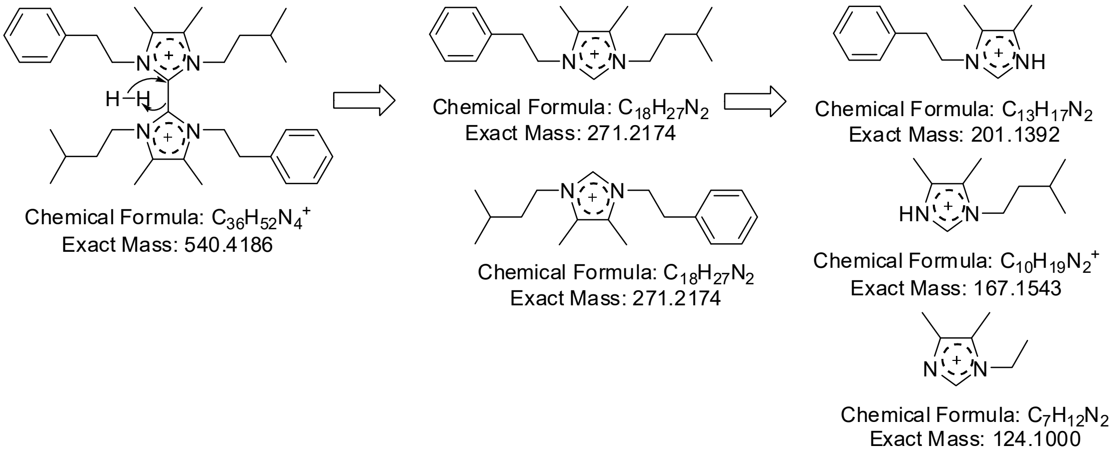

Figure S2. Schematic representation of feasible fragmentation pathway for Paenidigyamycin A (**1**) under HRESI-LC-MS conditions.

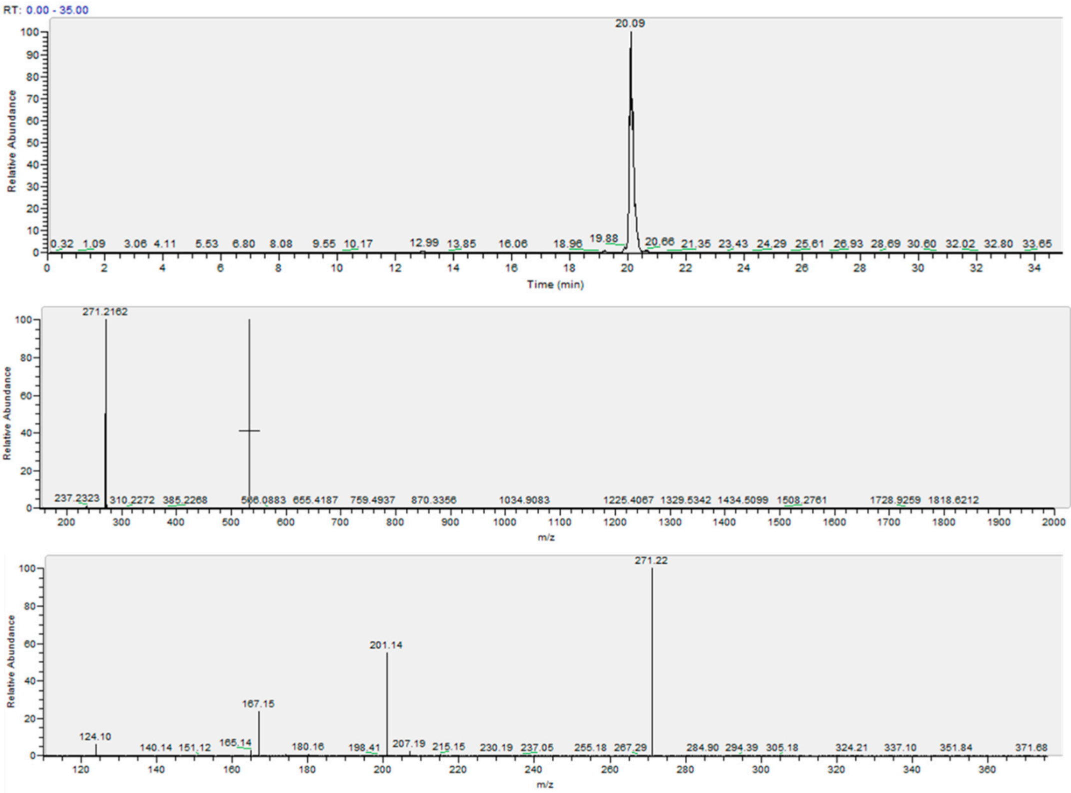

Figure S3. HRESI-LC-MS shows chromatogram that confirms the fragmentation pathway for Paenidigyamycin A (**1**).

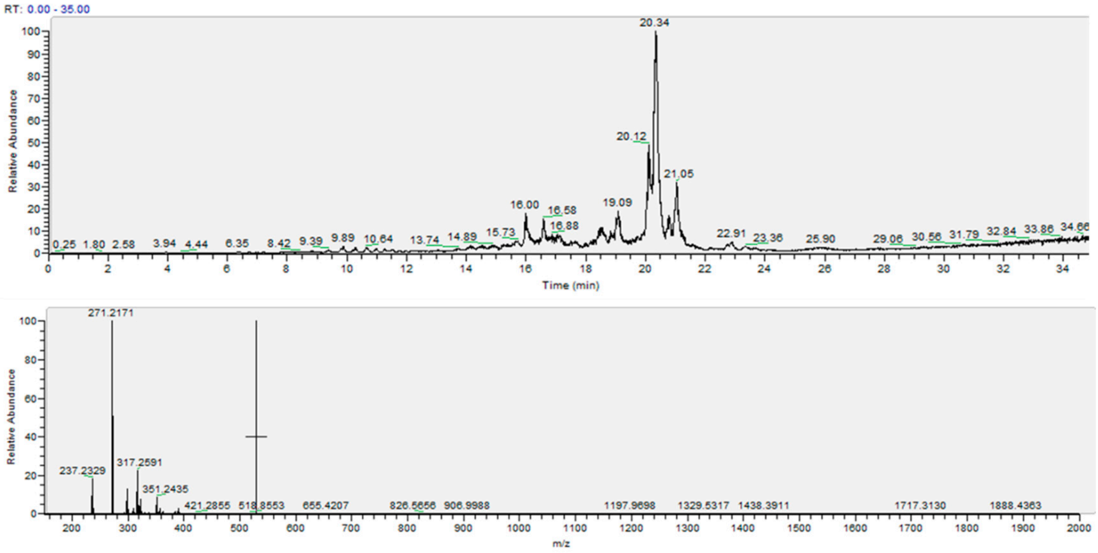

Figure S4. HRESI-LC-MS for crude FM extracts of strain DE2SH.

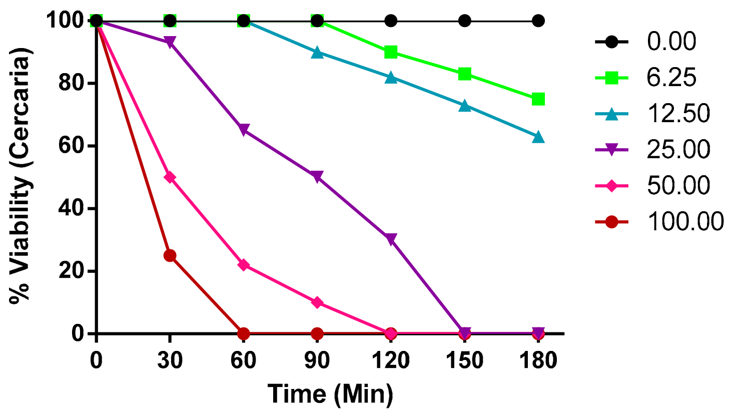

Figure S5. Effect of different concentrations of compound **1** on the viability of *Schistosoma mansoni* cercariae.

Table S1a. Full 1D and 2D NMR Spectroscopic data for Paenidigyamycin A (**1**) in CD_3OD, δ in ppm.

#	δ ^{13}C (ppm)	^{13}C mult	$δ_H$ Mult (J Hz)	^{1}H-^{1}H COSY	^{1}H-^{1}H TOCSY	NOESY	HMBC
1-N		-	-	-	-	-	
2	135.4	C	-	-	-	-	8, 10
3-N		-	-	-	-	-	
4	128.4	C	-	-	-	-	10, 6
5	128.4	C	-	-	-	-	8, 7
6	8.1	CH_3	2.27, s	-	-	10, 11	
7	8.1	CH_3	2.22, s	-	-	8, 9	
8	49.7	CH_2	4.40, t (6.8)	9	9	7, 2', 6', 9	9
9	36.7	CH_2	3.12, t (6.7)	8	8	8, 7, 2', 6'	8, 2', 6'
10	46.4	CH_2	4.03, m	11	11, 13, 14	13, 14, 11, 6	12, 11
11	39.5	CH_2	1.57, m	10	10, 13, 14	10, 6	13, 14, 10, 12
12	26.5	CH	1.48, n (6.8)	13, 14	13, 14		13, 14, 10
13	22.5	CH_3	0.96, d (6.6)	12	12, 11, 10	10	14
14	22.5	CH_3	0.96, d (6.6)	12	12, 11, 10	10	13
1'	137.8	C	-				9, 8, 3', 5'
2'	130.0	CH	7.10, m	3', 4'	3', 4'	8, 9	9, 4', 3', 5'
3'	130.0	CH	7.31, m	2'	2'		2', 6'
4'	128.4	CH	7.29, m	2', 6'	2', 6'		2', 6'
5'	130.0	CH	7.31, m	6'	6'		2', 6'
6'	130.0	CH	7.10, m	5', 4'	5', 4'	8, 9	9, 4', 3', 5'

Table S1b. Full 1D and 2D NMR Spectroscopic data for Paenidigyamycin A (**1**) in CD_3OD, δ in ppm (Cont.).

#	δ ^{13}C (ppm)	^{13}C mult	δ_H Mult (J Hz)	^1H-^1H COSY	^1H-^1H TOCSY	NOESY	HMBC
1'-N	-	-	-	-	-	-	-
2'	135.5	C	-	-	-	-	8', 10'
3'-N	-	-	-	-	-	-	-
4'	128.5	C	-	-	-	-	10', 6'
5'	128.5	C	-	-	-	-	8', 7'
6'	8.2	CH_3	2.31, s	-	-	10', 11'	
7'	8.0	CH_3	2.06, s	-	-	8', 9'	
8'	49.2	CH_2	4.33, t (6.9)	9'	9'	2'', 6'', 7', 9'	9'
9'	37.1	CH_2	3.04, t (6.9)	8'	8'	2'', 6'', 8', 7'	8'
10'	46.6	CH_2	4.13, m	11'	11', 13', 14'	13', 14', 12', 11', 6'	12', 11'
11'	39.5	CH_2	1.73, m	10'	10', 13', 14'	10', 6'	13', 14', 12', 10'
12'	26.9	CH	1.67, n (6.8)	13', 14'	13', 14'	10'	13', 14', 11', 10'
13'	22.5	CH_3	1.02, d (6.5)	12'	12', 11', 10'	10'	14'
14'	22.5	CH_3	1.02, d (6.5)	12'	12', 11', 10'	10'	13'
1''	137.8	C	-				3'', 5'', 9', 8'
2''	130.0	CH	7.10, m	3'', 4''	3'', 4''	9', 8'	4'', 3'', 5'', 9'
3''	130.0	CH	7.31, m	2''	2''		2'', 6''
4''	128.4	CH	7.29, m	2'', 6''	2'', 6''		2'', 6''
5''	130.0	CH	7.31, m	6''	6''		2'', 6'',
6''	130.0	CH	7.10, m	5'', 4''	5'', 4''	9', 8'	4'', 3'', 5'', 9'

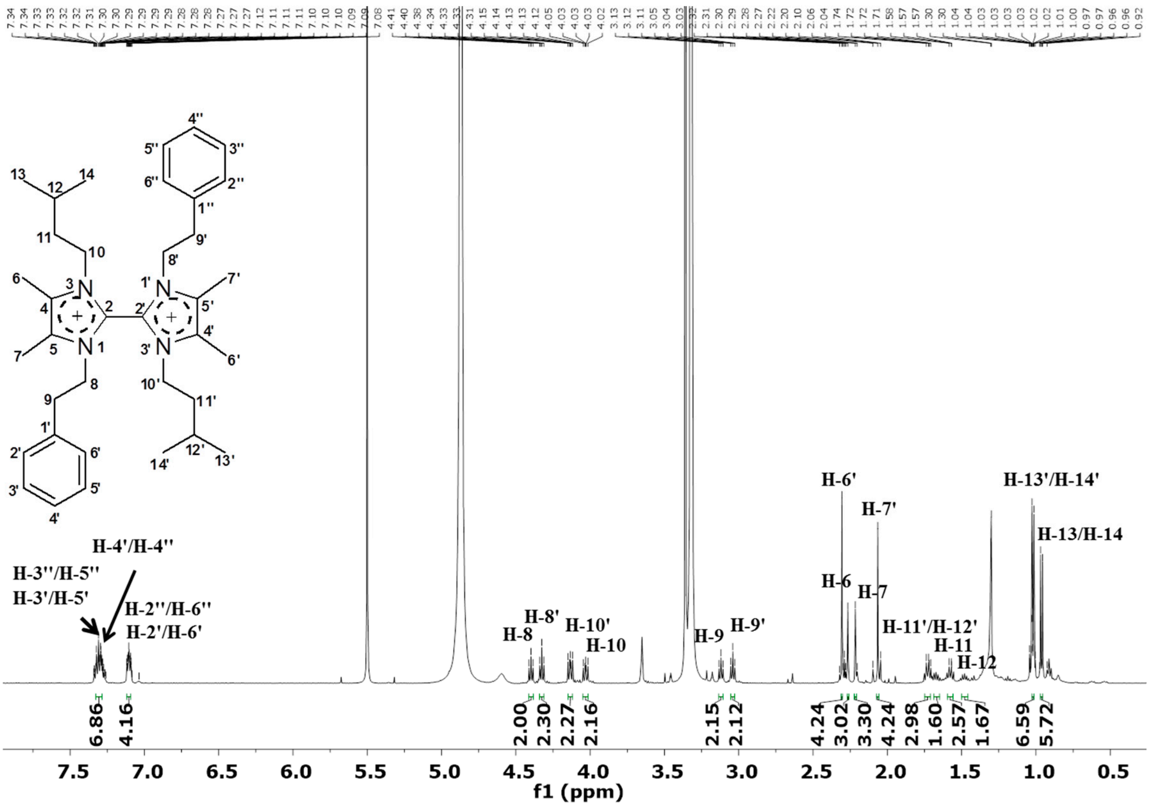

Figure S6. ^1H NMR spectrum (500 MHz) of Paenidigyamycin A (**1**) in CD_3OD.

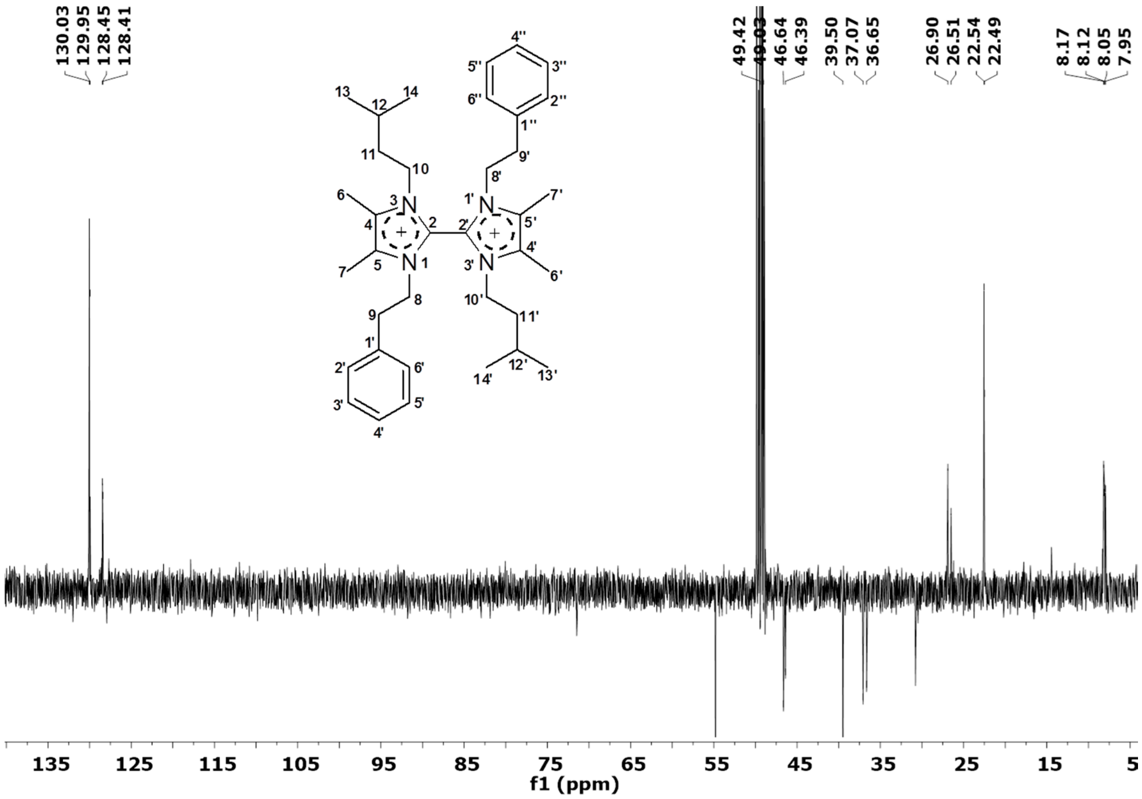

Figure S7. DEPT135° spectrum of Paenidigyamycin A (**1**) in CD_3OD.

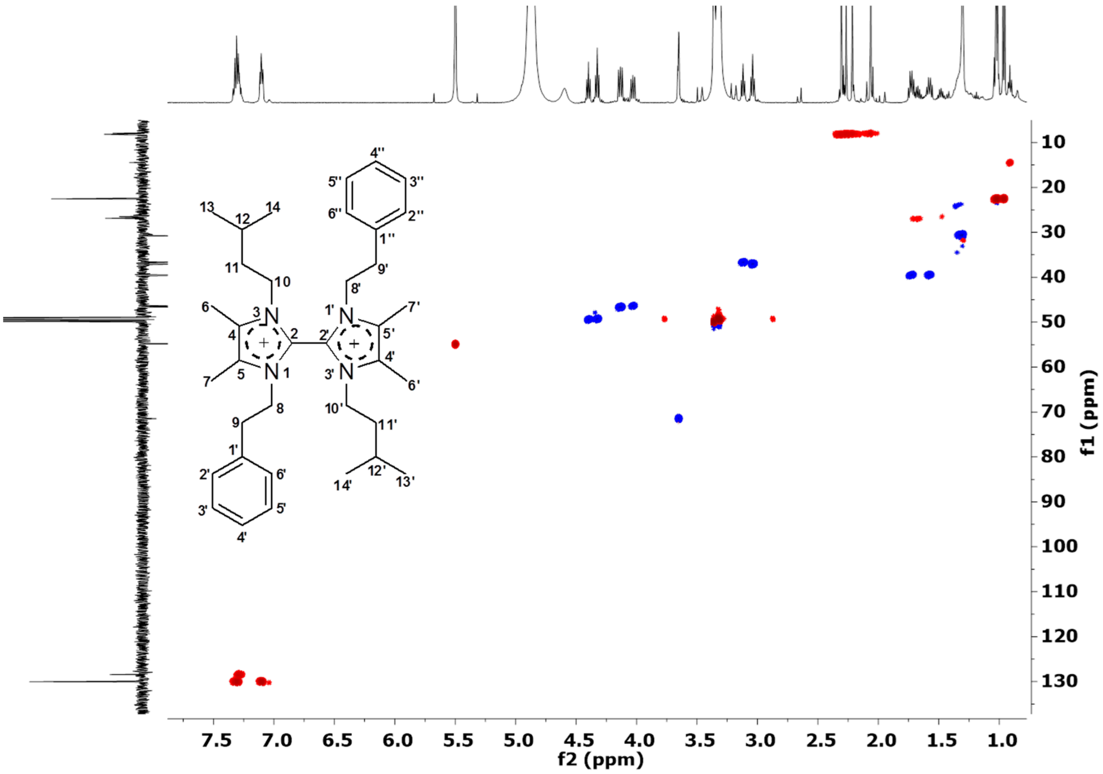

Figure S8. HSQC spectrum (500 MHz) of Paenidigyamycin A (**1**) in CD3OD.

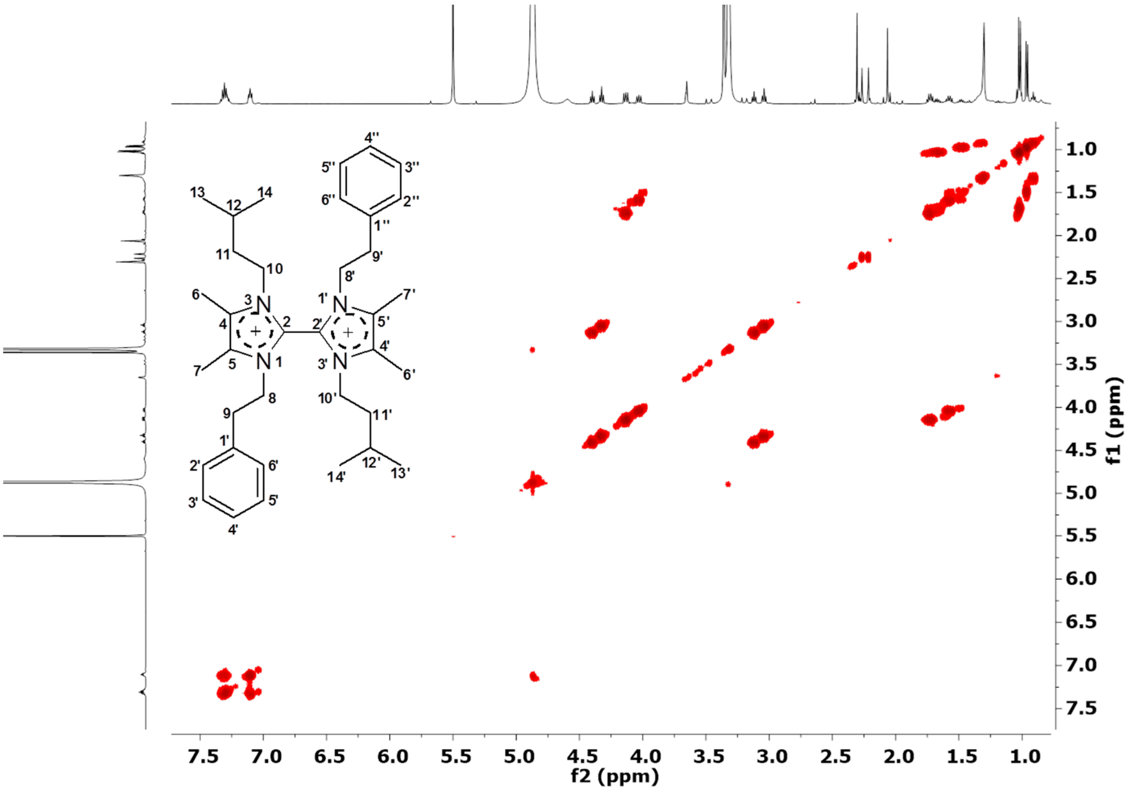

Figure S9. COSY spectrum (500 MHz) of Paenidigyamycin A (**1**) in CD_3OD.

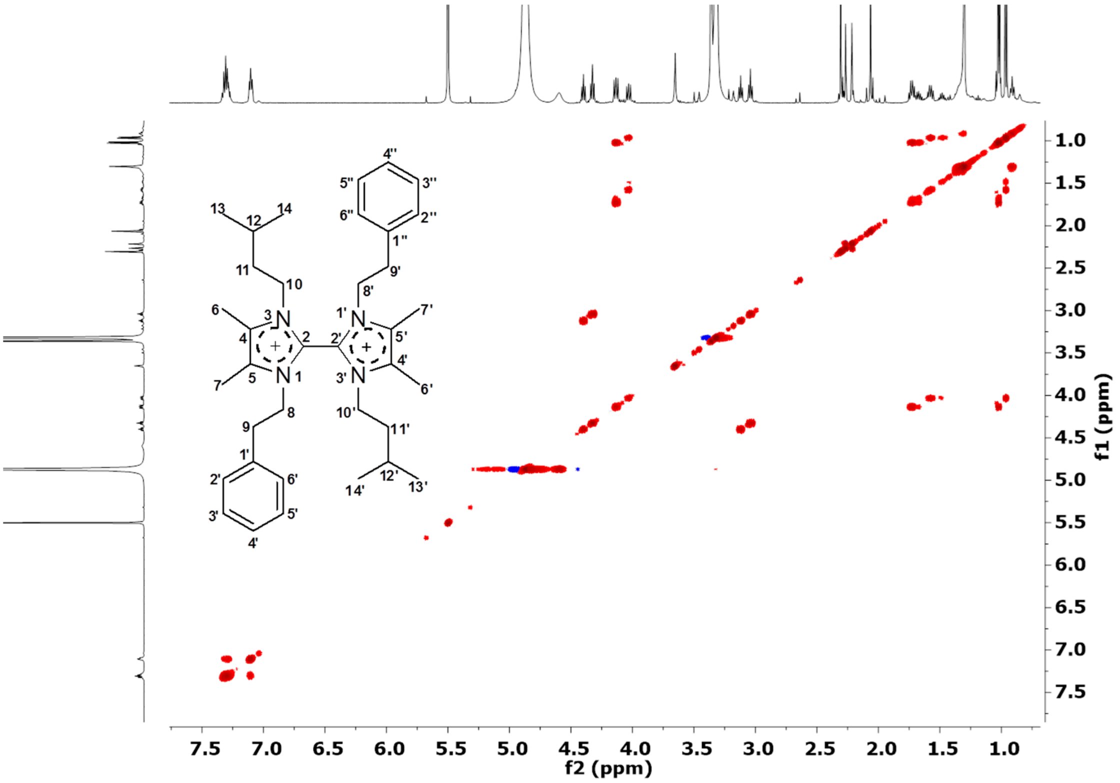

Figure S10. 2D-TOCSY spectrum (500 MHz) of Paenidigyamycin A (**1**) in CD_3OD.

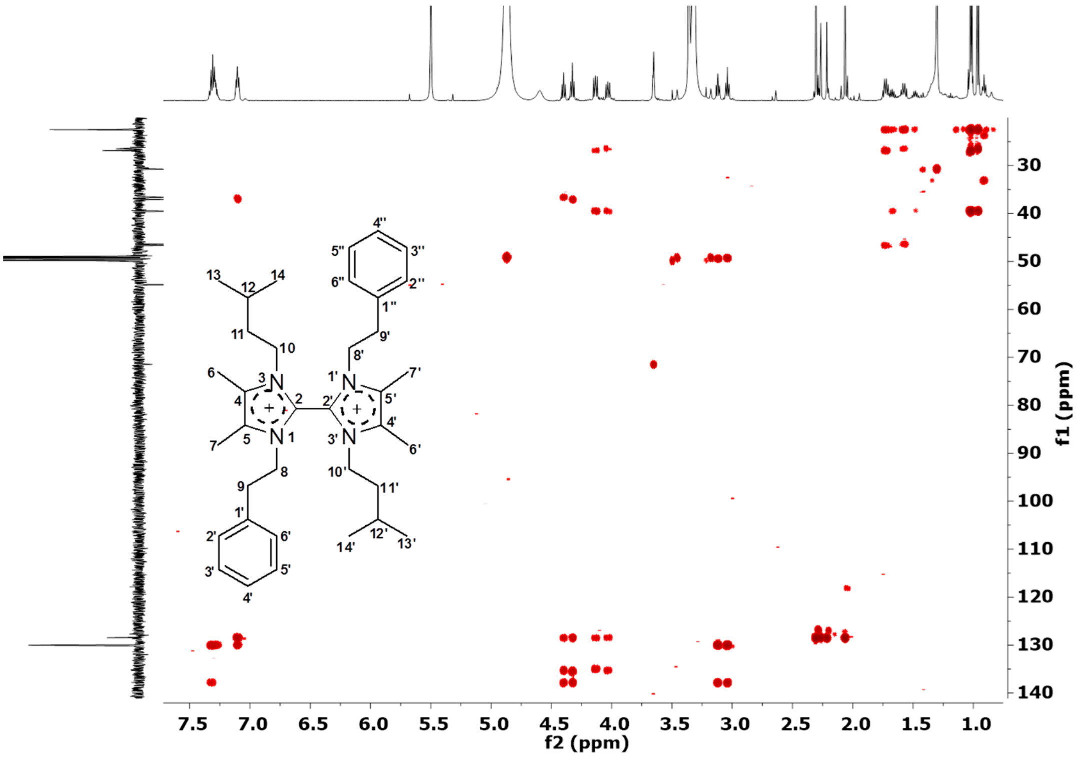

Figure S11. HMBC spectrum (500 MHz) of Paenidigyamycin A (**1**) in CD_3OD.

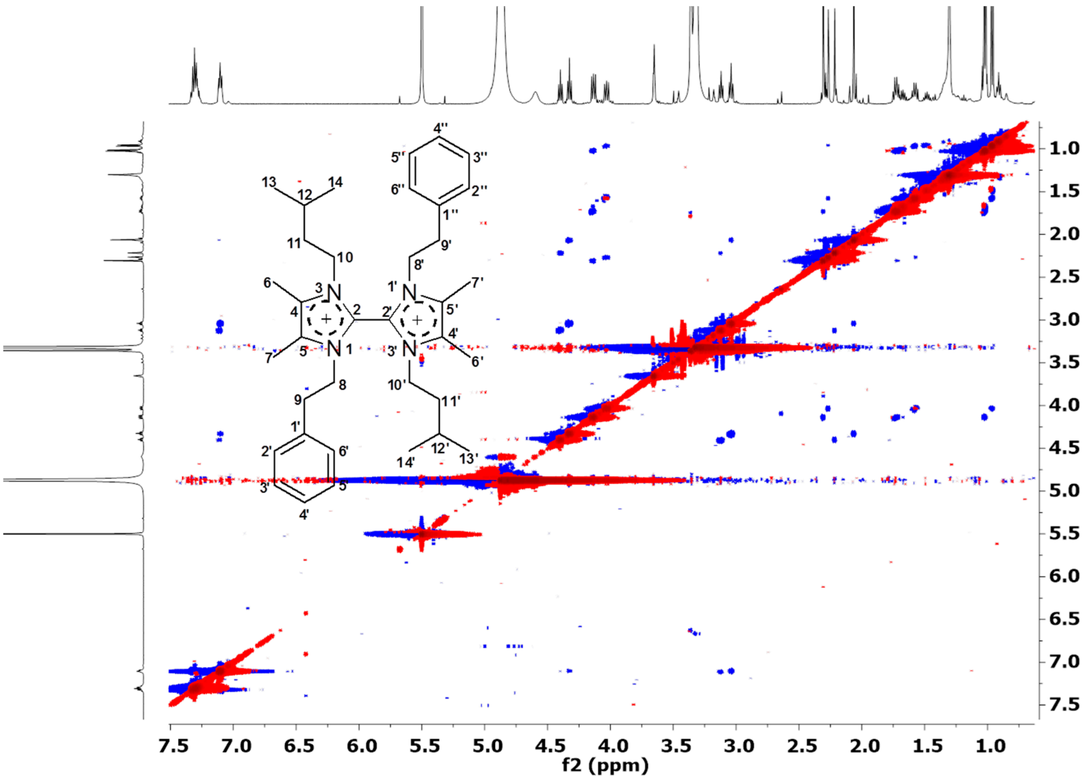

Figure S12. NOESY spectrum (500 MHz) of Paenidigyamycin A (**1**) in CD_3OD.

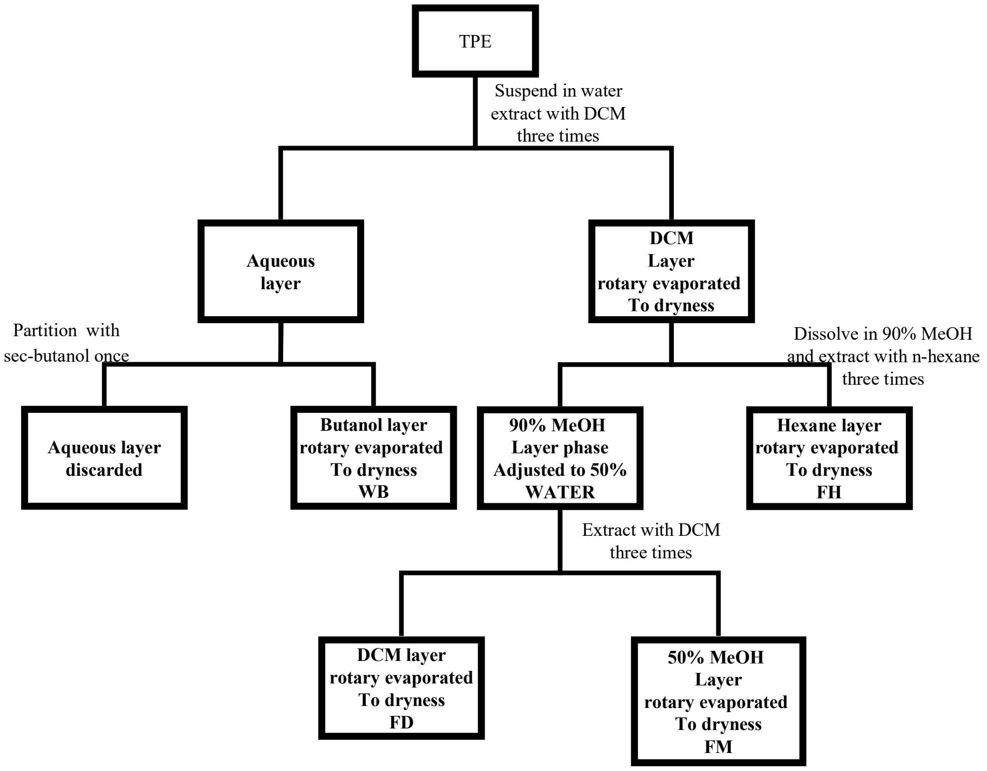

Figure S13. Modified Kupchan solvent partitioning of the crude extract of *Paenibacillus* sp. DE2SH gives FH, FD, FM, and WB fractions.

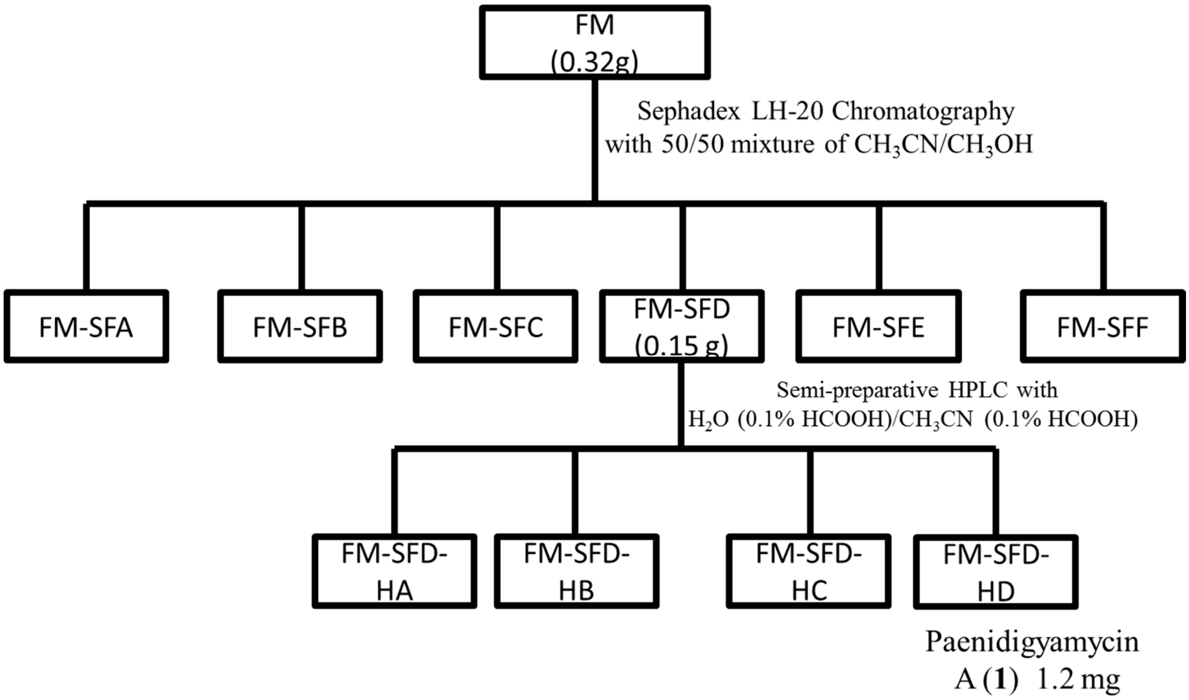

Figure S14. Sephadex LH-20 Chromatography of FM fraction followed by Semi-preparative HPLC gives pure Paenidigyamycin A (**1**).

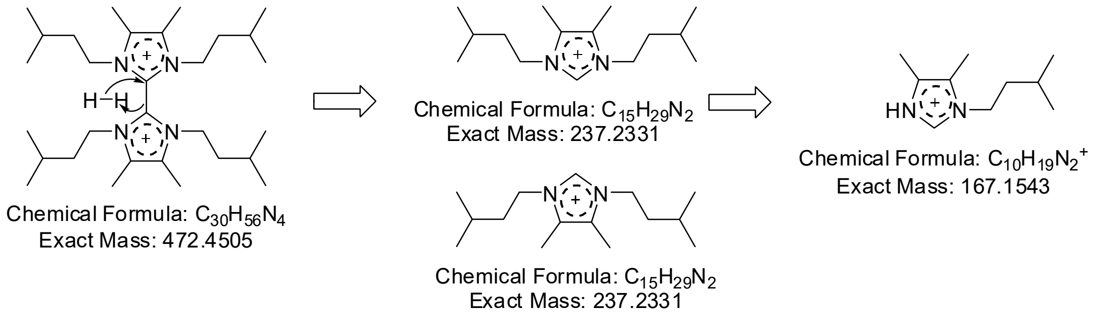

Figure S15. Schematic representation of a feasible fragmentation pathway for possible Paenidigyamycin A (**1**) analogue under HRESI-LC-MS-MS conditions.

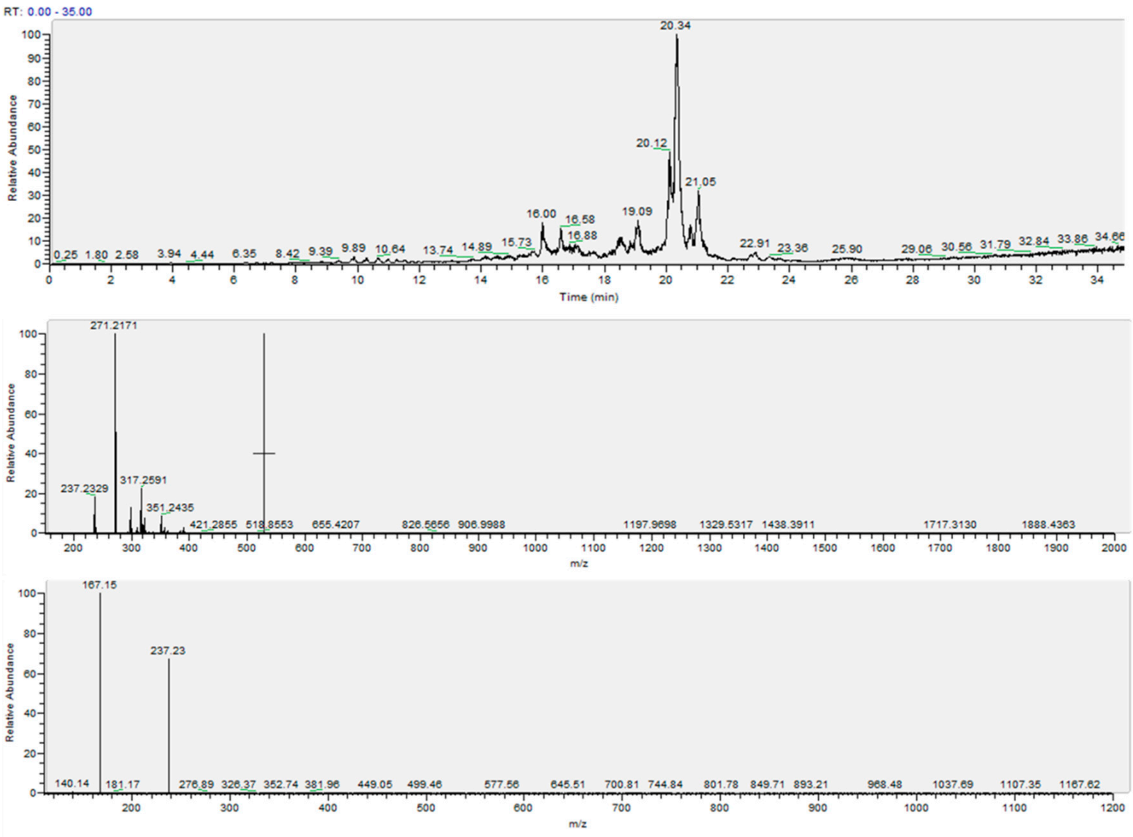

Figure S16. HRESI-LC-MS shows the possible presence of a Paenidigyamycin A (**1**) analogue.

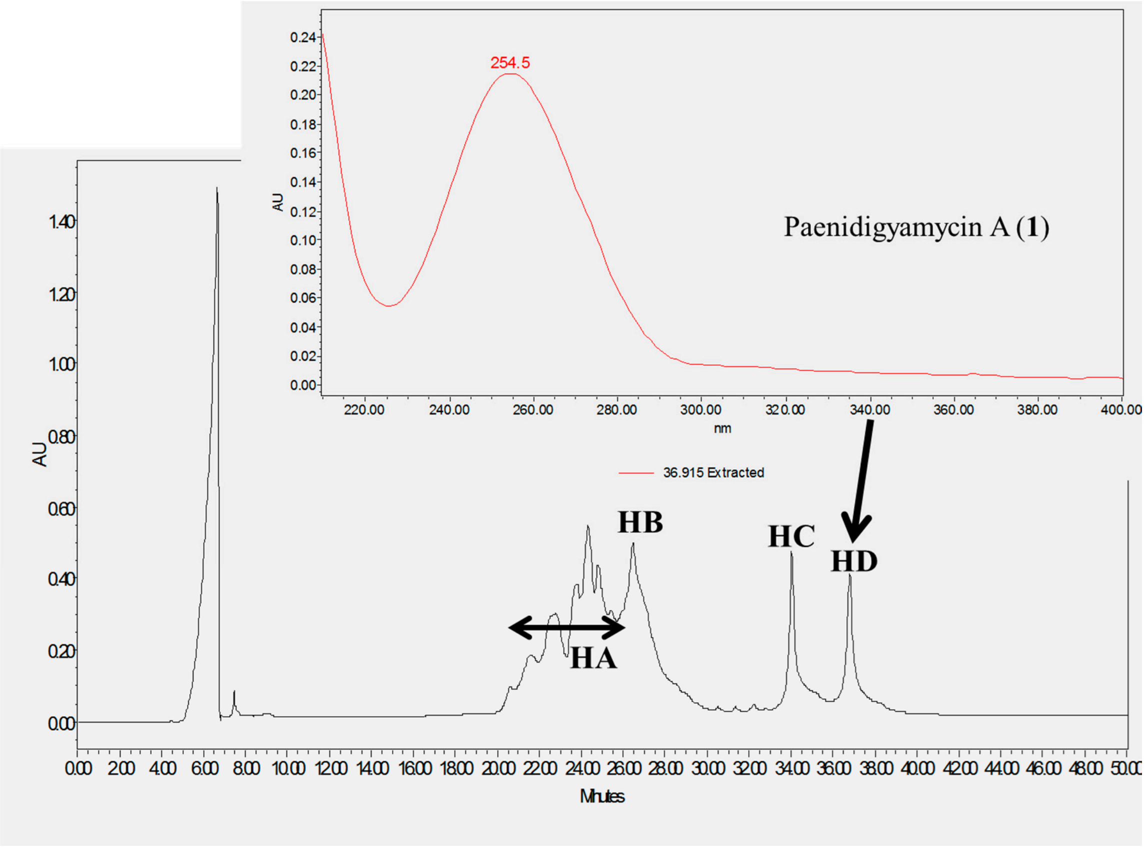

Figure S17. HPLC Chromatogram of Paenidigyamycin A (**1**) with UV profile.
